# A 12-Week Pilot Study Comparing High-Intensity Interval Training and Peripheral Heart Action Training on ISAK-Based Anthropometric Outcomes and Perceived Psychophysical Well-Being in Young Adults

**DOI:** 10.3390/sports14030102

**Published:** 2026-03-04

**Authors:** Felice Di Domenico, Rosario Ceruso, Gaetano Raiola, Sara Aliberti, Giovanni Esposito

**Affiliations:** Research Center of Physical Education and Exercise, Pegaso University, 80143 Naples, Italy; flcdidomenico@gmail.com (F.D.D.); rosario.ceruso@univr.it (R.C.); s.aliberti17@gmail.com (S.A.); giovanniesposito410@gmail.com (G.E.)

**Keywords:** high-intensity interval training, peripheral heart action, body composition, anthropometry (ISAK), perceived well-being

## Abstract

Background: High-Intensity Interval Training (HIIT) and Peripheral Heart Action (PHA) are widely used training modalities, but comparative longitudinal data using standardized anthropometric methods remain limited. Purpose: To compare within-group changes over 12 weeks of HIIT and PHA training on body composition and perceived psychophysical well-being in moderately active young adults. Methods: Twenty-four adults (12 males, 12 females; age 30.9 ± 3.5 years) were allocated to either HIIT or PHA in a non-randomized pilot study, based on training schedule availability and previous training routine, which may introduce selection bias. Training was performed three times per week for 12 weeks. Body composition was assessed using standardized ISAK anthropometry. Data were analyzed using linear mixed-effects models. Results: Significant effects of Time were found for body mass, BMI, sum of skinfolds, waist circumference, and endomorphy (all *p* < 0.05). Significant Time × Group interactions were observed for BMI, sum of skinfolds, waist circumference, and endomorphy (*p* < 0.05), indicating different adaptation patterns. HIIT showed greater reductions in selected skinfolds and higher perceived performance improvement (*p* < 0.001), whereas PHA showed greater increases in arm circumferences and mesomorphy (*p* < 0.01). Conclusions: Within-group improvements were observed in anthropometric/body composition indicators over time, with distinct longitudinal adaptation patterns between HIIT and PHA.

## 1. Introduction

Structured exercise represents one of the main non-pharmacological interventions to improve health and well-being in the general population, with well-documented effects on cardiorespiratory fitness, metabolism, and psychological health [1]. In recent years, scientific interest has increasingly focused on time-efficient training methods that can be implemented in real-world settings such as personal training studios or individualized exercise programs. In time-constrained adults, individualized and time-efficient training prescriptions may improve the likelihood of sustained participation by reducing perceived barriers and optimizing adherence. From a public-health perspective, such approaches can support cardiometabolic and morphological health improvements while remaining compatible with real-world schedules, which is particularly relevant in settings characterized by low physical activity levels and limited available time. Among these, High-Intensity Interval Training (HIIT) has received particular attention, with numerous studies confirming its effectiveness in improving cardiorespiratory fitness (VO_2_max) and body composition, often with a lower time commitment compared with moderate-intensity continuous training [2,3]. From a physiological perspective, HIIT induces marked metabolic stress, promotes the activation of pathways responsible for mitochondrial biogenesis such as PGC-1α, and increases post-exercise oxygen consumption (EPOC), thereby contributing to greater overall energy expenditure [4,5].

In parallel, increasing attention has been directed toward circuit-based training protocols characterized by alternating exercises with short recovery periods and moderate-to-high intensity. Although these methods do not reach the intensity peaks of HIIT, they maintain an elevated heart rate and require continuous metabolic engagement [6]. Total-body circuit training has been shown to improve aerobic capacity, muscular strength, and body composition, with a generally high tolerability profile [7,8]. The Peripheral Heart Action (PHA) protocol, which involves the alternation of upper- and lower-body exercises, promotes a sustained increase in heart rate through the hemodynamic demand associated with continuous redistribution of blood flow between body segments [9], making it suitable also for health-oriented, non-athletic populations.

From a psychological perspective, several studies have shown that affective responses and perceived exertion differ substantially between high-intensity protocols and moderate or interval-circuit approaches, influencing exercise adherence and long-term sustainability [10]. In particular, although HIIT is effective, it may be perceived as more demanding and less pleasant, especially by less trained individuals [11,12]. In contrast, circuit training and moderate-intensity exercise tend to elicit more positive affective responses, thereby facilitating continued participation [13].

A major limitation of applied exercise research concerns the lack of standardization in anthropometric measures used to assess changes in body composition. In many studies, such changes are estimated using indirect indicators such as BMI or bioelectrical impedance, which, despite their practicality, present substantial limitations in terms of accuracy, sensitivity to change, and validity when the goal is to assess morphological adaptations induced by exercise [14,15]. In particular, BMI does not distinguish between fat mass and fat-free mass and cannot detect changes in fat distribution, making it poorly sensitive to adaptations typically induced by combined or high-intensity training programs. Similarly, bioelectrical impedance is strongly influenced by hydration status, body temperature, and environmental variables, resulting in high intra-individual variability that limits its use in longitudinal and comparative studies [15,16]. In contrast, standardized anthropometric protocols, such as those proposed by the International Society for the Advancement of Kinanthropometry (ISAK), allow for a more accurate and reproducible assessment of subcutaneous adiposity, body circumferences, and somatotype, enabling the detection of qualitative changes in body composition that are often missed by indirect methods [17,18]. The adoption of these protocols significantly improves the internal validity of studies and facilitates comparisons across investigations.

Despite the widespread use of the Peripheral Heart Action (PHA) protocol in health-oriented exercise and personal training, the scientific literature directly comparing its effects with those of High-Intensity Interval Training (HIIT) remains limited. From a mechanistic perspective, HIIT and PHA are expected to elicit partially different physiological stimuli. HIIT induces pronounced metabolic stress and cardiometabolic adaptations through repeated high-intensity bouts, whereas PHA maintains a sustained cardiovascular load via alternating upper- and lower-body exercises, providing a more continuous neuromuscular stimulus. Consequently, the two modalities may lead to distinct anthropometric and perceptual adaptation trajectories. Most comparative studies on cardiorespiratory exercise focus on continuous training versus HIIT, while hybrid methods such as PHA are largely neglected. Moreover, available studies often present methodological limitations, including heterogeneity of training protocols, poor standardization of anthropometric measurements, and limited consideration of perceptual variables related to psychophysical well-being and exercise tolerance [10,11]. The relevance of perceptual variables and movement awareness has been highlighted in recent methodological contributions showing that different training approaches can influence not only performance but also subjective perception, self-awareness, and the quality of the movement experience [19,20]. However, these dimensions remain insufficiently investigated in exercise studies focused on well-being in non-athletic adult populations. Consequently, it is still unclear whether and to what extent PHA may represent, in young and moderately active adults, an effective and sustainable alternative to HIIT for improving body composition and perceived well-being, especially when such effects are evaluated using rigorous measurement tools in real-world personal training settings.

This pilot study was therefore designed to compare the effects of 12 weeks of HIIT and PHA on changes in fat mass assessed by ISAK-based skinfold measurements, as well as on BMI, somatotype, and body circumferences. In addition, subjective perceptions of psychophysical well-being were assessed at the end of the intervention in order to explore the relationship between anthropometric changes and perceived well-being. These data are intended to evaluate the feasibility of the protocols and the quality of adherence to the two methods, in preparation for future studies with larger samples and randomized allocation. The study does not aim to identify a universally superior protocol, but rather to explore potential differences in adaptation patterns and subjective exercise perception. Given the non-randomized allocation based on availability and prior training routine, this pilot study is exploratory in nature and does not support causal inference. This non-randomized allocation may have introduced confounding due to factors that could systematically differ between groups, including (i) previous exposure to HIIT- or circuit-style training (training novelty), (ii) baseline fitness and exercise tolerance, (iii) motivation and training preference, and (iv) schedule-related constraints that may correlate with lifestyle and habitual physical activity patterns. These factors may influence responsiveness and adaptation trajectories, thereby limiting causal interpretation of between-group differences. Therefore, baseline comparability cannot be assumed, and the results should be interpreted as preliminary.

## 2. Materials and Methods

### 2.1. Study Design

This study was conducted as a 12-week non-randomized longitudinal pilot trial with repeated measures. Participants were allocated to either the HIIT or the PHA group based on training schedule availability and their previous training pathway, resulting in two parallel intervention arms. Therefore, selection bias cannot be excluded, and findings should be interpreted as exploratory.

**Figure 1:**
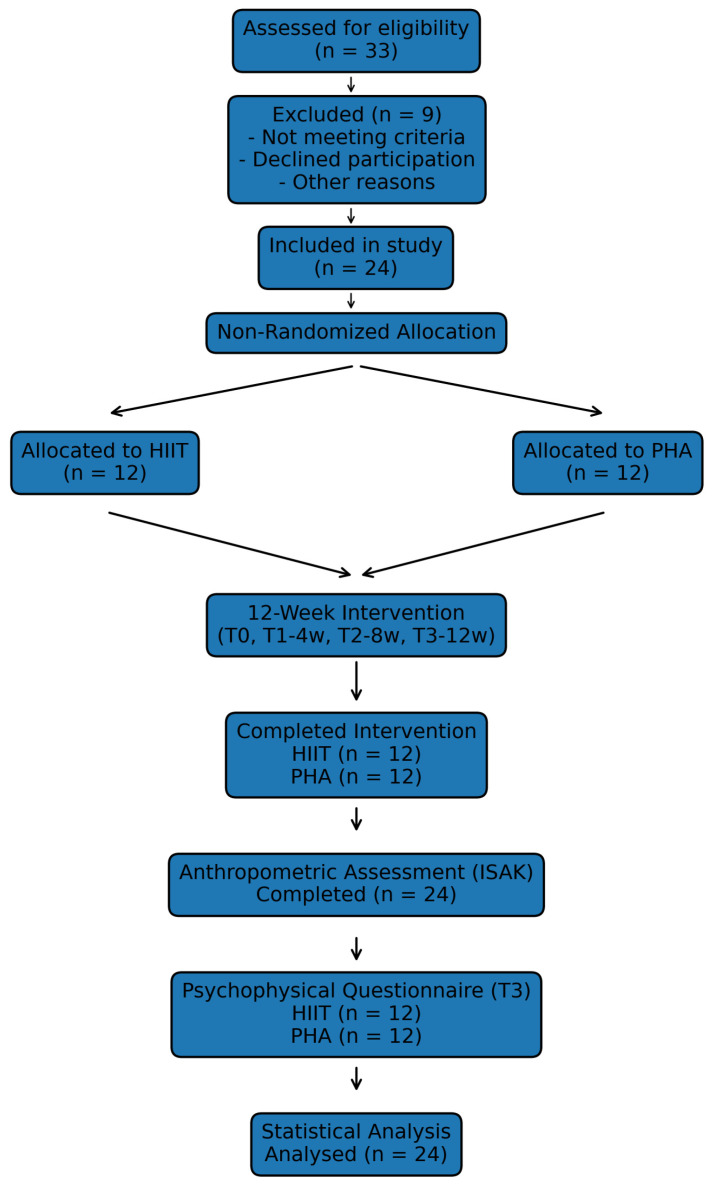
Study workflow from recruitment to final statistical analysis. A total of 33 individuals were assessed for eligibility; 9 were excluded. Twenty-four participants were included and allocated to HIIT (*n* = 12) or PHA (*n* = 12) based on schedule availability and previous training pathway. All participants completed the 12-week intervention (T0–T3), anthropometric assessment, and final statistical analysis.

The study workflow, including recruitment (*n* = 33), exclusions (*n* = 9), non-randomized allocation (HIIT *n* = 12; PHA *n* = 12), intervention period (T0–T3), outcome assessments, and final analysis (*n* = 24), is illustrated in Figure 1.

Measurements were performed at four time points: baseline (T0), after 4 weeks (T1), 8 weeks (T2), and at the end of the intervention (12 weeks, T3). At each time point, standardized anthropometric assessments were collected, while psychophysical well-being was evaluated at the end of the intervention.

Both groups completed three supervised training sessions per week throughout the 12-week period, following their assigned protocols. Participants were instructed not to engage in any additional structured physical activity outside the intervention and to maintain their habitual lifestyle and dietary patterns. Training adherence to intensity prescriptions was monitored continuously via heart rate (Polar H9 and Polar Flow). Because physical activity outside the intervention was not objectively monitored, differences in habitual or additional activity may have contributed to the magnitude of observed anthropometric changes and increased variability between participants. This limitation is inherent to real-world training studies and should be considered when interpreting between-group differences and results should be interpreted accordingly, particularly in small-sample pilot designs.

This repeated-measures design allowed the investigation of within-group changes over time and between-group differences in adaptation patterns in a real-world personal training setting.

### 2.2. Participants

The sample consisted of 24 adults (12 males and 12 females; mean age 30.9 ± 3.5 years; BMI 24.5 ± 3.3 kg·m^−2^) recruited through convenience sampling from clients of a personal training studio located in the province of Salerno (Italy).

Inclusion criteria were: age between 18 and 40 years, regular participation in physical activity for at least 12 months, absence of medical conditions limiting physical exercise, and willingness to participate in a 12-week supervised training program.

Participants were allocated to the HIIT or PHA group according to their availability and previous training routine, resulting in a non-randomized design. Allocation was based on (i) training schedule availability (session time slots offered by the studio) and (ii) participants’ previous training pathway (self-reported primary routine in the preceding months). Individuals were assigned to the group that best matched their availability while minimizing abrupt deviations from their habitual training structure. Previous exposure to HIIT- or PHA-like training was not formally quantified using a standardized instrument; therefore, differential responsiveness due to training novelty cannot be excluded and should be considered a potential confounder. Baseline anthropometric characteristics and between-group comparisons are reported in Table 1. No statistically significant differences were observed between groups for the primary anthropometric variables at baseline. However, given the non-randomized allocation procedure, baseline comparability cannot be assumed a priori, and findings should be interpreted with caution.

The present study is part of the research project entitled “Psychophysical perception and body awareness in school and sports contexts through observational and non-invasive tools”, approved by the Ethics Committee of Pegaso University (Prot./E 004726, dated 15 July 2025). All participants provided written informed consent prior to participation.

### 2.3. Nutritional Control

To reduce potential confounding due to changes in dietary habits, participants were instructed to maintain their usual diet throughout the intervention and not to undertake any nutritional program aimed at modifying body weight or body composition.

Dietary intake was monitored using a 3-day food diary (two weekdays and one weekend day), collected at baseline (T0) and at the end of the intervention (T3). The diaries were used to estimate daily energy intake and macronutrient distribution. However, as dietary monitoring relied on self-reported records, adherence and accuracy cannot be fully verified.

### 2.4. Training Protocols

The intervention lasted 12 weeks, with three training sessions per week, each lasting approximately 50–60 min. All sessions were supervised by qualified exercise physiologists (kinesiologists) with experience in high-intensity and circuit-based training, who ensured correct exercise execution and continuously monitored training loads and target intensities according to the protocol.

All training loads, work-to-rest ratios, and intensity targets were applied uniformly within each group. Exercise selection was adapted only when necessary due to orthopedic or technical limitations. Exercise substitutions were required in 3 participants (HIIT *n* = 2; PHA *n* = 1), resulting in a total of 3 substituted exercises across the intervention. Substitutions were restricted to functionally equivalent movements targeting the same primary muscle groups and intended intensity. Given the limited number of substitutions, intervention fidelity was considered largely preserved, although minor variability in the training stimulus cannot be excluded.

#### 2.4.1. Training Intensity Monitoring

During all training sessions, exercise intensity was monitored using a Polar H9^®^ chest-strap heart rate monitor (Polar Electro Oy, Kempele, Finland), which allowed continuous recording of heart rate and ensured adherence to the predefined internal load target ranges:

- HIIT: 80–95% of HR_max
- PHA: 70–80% of HR_max

Heart rate data (beats per minute, % HR_max, and time spent in intensity zones) were synchronized after each session using the Polar Flow application. Table 2 summarizes the physiological parameters monitored during the training sessions, the data collection methods, and their respective applications. Continuous heart rate monitoring provided objective assessment of internal training load through % HR_max and time spent within the predefined intensity zones, ensuring adherence to the intended stimulus of each protocol. However, compliance with the recommendation to refrain from additional structured physical activity outside the intervention was not objectively monitored and should be considered a limitation.

#### 2.4.2. HIIT Protocol

The HIIT protocol consisted of a high-intensity functional circuit structured as follows:

- Six exercises, each performed for 30 s of work followed by 30 s of passive recovery
- Four total rounds, with 1 min of recovery between rounds
- Target intensity maintained between 80 and 95% of HR_max

The exercises included combinations of battle rope, modified burpees, kettlebell swings, TRX rows, cycling sprints, and jump rope.

The total duration of the HIIT main set was approximately 25 min, resulting in a total session duration of approximately 45–50 min, including:

- 10 min warm-up (50–60% HR_max)
- 24 min HIIT circuit
- 10 min cool-down

**Figure 2:**
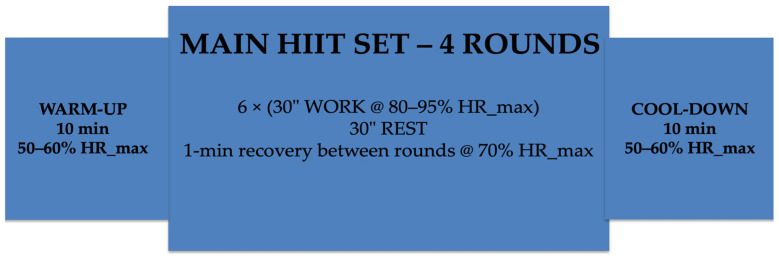
Schematic representation of the HIIT session structure (total duration: 50 min). The session included a 10-min warm-up (50–60% HR_max), followed by a main set composed of four rounds of six exercises (30 s work at 80–95% HR_max interspersed with 30 s rest), with 1-min recovery between rounds (~70% HR_max), and a 10-min cool-down (50–60% HR_max).

The detailed structure of the HIIT protocol is summarized in Table 3. A schematic representation of the HIIT session structure is presented in Figure 2.

#### 2.4.3. PHA Protocol

The PHA protocol followed the structure below:

- Six exercises alternating upper- and lower-body movements
- 40 s of work/20 s of rest
- Four total rounds
- 1 min of recovery between rounds
- Target intensity: 70–80% of HR_max

This configuration was designed to maintain a stable cardiovascular load without the intensity peaks typical of HIIT, while simultaneously optimizing the muscular–endurance component of the training stimulus.

**Figure 3:**
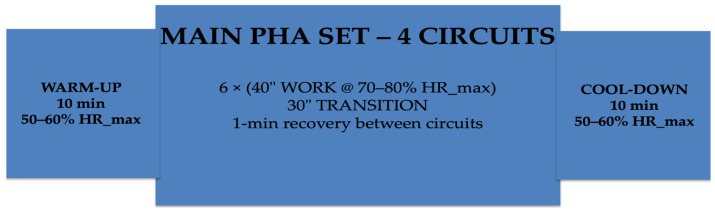
Schematic representation of the PHA session structure (total duration: 55 min). The session included a 10-min warm-up (50–60% HR_max), followed by a main set composed of four circuits of six stations (40 s work at 70–80% HR_max with 20 s transition between stations), with 1-min recovery between circuits, and a 10-min cool-down (50–60% HR_max).

The detailed structure of the PHA training protocol is presented in Table 4. A schematic representation of the PHA session structure is presented in Figure 3.

### 2.5. Anthropometric Measurements (ISAK Protocol)

Anthropometric assessments were performed by an ISAK Level 1-certified operator following the standardized procedures of the International Society for the Advancement of Kinanthropometry (ISAK) at four time points: baseline (T0), after 4 weeks (T1), 8 weeks (T2), and at the end of the intervention (T3).

Skinfold thickness was measured at the triceps, subscapular, biceps, iliac crest (suprailiac), supraspinale, abdominal, thigh, and calf sites using a Harpenden Skinfold Caliper^®^ (Baty International, Burgess Hill, UK). Each site was measured twice in rotational order; if the difference between the two measurements exceeded 5%, a third measurement was taken. The median value was used for analysis, in accordance with ISAK guidelines.

Body circumferences were measured at the waist, hip, relaxed arm, flexed arm, thigh, and calf. Somatotype (endomorphy, mesomorphy, and ectomorphy) was calculated according to the Carter and Heath method. Fat mass was estimated using the anthropometric fractionation equation proposed by Kerr [21] for adipose tissue mass, while skeletal muscle mass was estimated using the predictive equation proposed by Lee et al. [22]. These equations were implemented within the ISAK-certified anthropometric software used for data processing, ensuring standardized computation procedures.

### 2.6. Psychophysical Well-Being Questionnaire

At the end of the intervention (T3), participants completed a questionnaire composed of three domains, each including two items, rated on a 5-point Likert scale (1–5), to assess perceived psychological well-being, physical/functional well-being, and perceived performance improvement.

The domains were:

- Psychological well-being
  1. I feel psychologically better compared with before the intervention.
  2. I perceived an increase in my motivation during the training program.
- Physical/functional well-being
  1. I perceive an improvement in my general physical well-being.
  2. I feel more capable and functional in performing daily activities.
- Perceived performance improvement
  1. I feel that my overall physical performance has improved.
  2. I perceive myself as stronger, faster, or more resistant than before the intervention.

The questionnaire was administered only at the end of the intervention (T3); therefore, analyses were limited to between-group comparisons.

### 2.7. Statistical Analysis

Statistical analyses were performed using JASP (version 0.95.4). Baseline between-group comparisons (HIIT vs. PHA at T0) for the main anthropometric variables were conducted using independent-samples *t*-tests when normality assumptions were met, or Mann–Whitney U tests when normality was violated. These analyses were performed to describe initial group characteristics; however, due to the non-randomized allocation, baseline equivalence cannot be interpreted as evidence of true comparability.

Normality was assessed using the Shapiro–Wilk test. When significant deviations from normality were detected (*p* < 0.05), non-parametric tests were applied. Accordingly, statistical procedures (parametric or non-parametric) were selected based on data distribution.

To evaluate within-group changes between T0 and T3, paired-sample *t*-tests were used; when normality assumptions were violated, the Wilcoxon signed-rank test was applied. Between-group differences in final values (T3) and in changes (Δ = T3 − T0) were analyzed using independent-samples *t*-tests. Effect sizes were calculated for all comparisons, reporting Cohen’s d for parametric tests and r for non-parametric tests.

Longitudinal changes were analyzed using Linear Mixed-Effects Models (LMMs), including Time, Group, and their interaction as fixed effects and participant as a random effect. Post hoc comparisons of the estimated marginal means were corrected for multiple testing using the Bonferroni method. Because allocation was non-randomized, we prioritized longitudinal inference via LMMs across T0–T3. Change-score (Δ) comparisons are reported descriptively and interpreted cautiously, as they may be influenced by baseline differences.

Perceived psychophysical well-being at T3 was analyzed using the three questionnaire domains. Domain scores were calculated as the mean of the corresponding items. Between-group comparisons (HIIT vs. PHA) were performed using the Mann–Whitney U test, given the ordinal nature of the data and the small sample size. Where appropriate, effect sizes (r) were also reported.

## 3. Results

Baseline between-group comparisons were conducted for the main anthropometric variables, and no statistically significant differences were observed (see Table 1).

Within-group changes from T0 to T3 were observed in several anthropometric variables in both groups; however, due to the non-randomized design and absence of a non-exercise control group, these changes cannot be conclusively attributed to the interventions (Table 5). Changes in anthropometric and body composition variables between baseline (T0) and the end of the intervention (T3) are reported in Table 5 (within- and between-group differences). Data analysis revealed significant improvements within both groups, although with different patterns depending on the variable considered.

In the HIIT group, significant reductions were observed in several skinfolds, including triceps, subscapular, suprailiac, supraspinale, abdominal, thigh, and calf (all *p* < 0.01), accompanied by large effect sizes. Significant reductions were also found for waist and hip circumferences and BMI (*p* < 0.001), indicating an overall improvement in body composition. Regarding somatotype, the HIIT group showed a significant decrease in endomorphy and significant increases in both mesomorphy and ectomorphy (*p* < 0.01).

In the PHA group, significant reductions were found in several skinfolds, including biceps, suprailiac, supraspinale, abdominal, thigh, and calf (all *p* < 0.01), generally with large effect sizes. In addition, the PHA group showed significant increases in arm circumferences, both relaxed and flexed (*p* < 0.01), suggesting a positive muscular adaptation. Similarly to the HIIT group, significant reductions were observed in waist and hip circumferences and BMI (*p* < 0.001). The somatotype analysis also revealed a decrease in endomorphy and increases in mesomorphy and ectomorphy.

Between-group comparisons of changes (Δ = T3 − T0) revealed significant differences in favor of the HIIT protocol for selected skinfolds, particularly triceps and supraspinale, whereas the PHA protocol produced greater increases in arm circumferences and in the mesomorphic component of the somatotype. For most of the remaining variables, no significant between-group differences were observed, suggesting comparable patterns of change across protocols in this sample of moderately active young adults.

Between-group Δ comparisons were not formally adjusted for baseline values; therefore, baseline imbalance may influence these findings. Given the non-randomized allocation, Δ comparisons should be considered exploratory rather than robust estimates of causal between-group effects. In this context, LMM Time × Group interactions provide a less biased indication of differential trajectories across T0–T3. This is because LMMs incorporate all repeated measurements across time, account for within-subject correlation, and model trajectories rather than relying on simple change scores, thereby reducing sensitivity to baseline imbalance and providing a more robust exploratory characterization of adaptation patterns. It should be noted that effect sizes should be interpreted cautiously given the small sample size and pilot nature of the study, as large estimates in small samples may reflect statistical instability rather than precise magnitude of effect.

Longitudinal changes in anthropometric and body composition variables were analyzed using linear mixed-effects models (LMMs), including Time (T0–T3), Group (HIIT vs. PHA), and their interaction (Time × Group) as fixed effects, and participant as a random effect (random intercept). Post hoc comparisons of the estimated marginal means were adjusted for multiple testing using the Bonferroni correction.

A significant main effect of Time was observed for body mass, BMI, sum of skinfolds, waist and hip circumferences, and the endomorphic and mesomorphic components of the somatotype (*p* < 0.05–0.001), indicating an overall improvement in body composition across the 12-week intervention, regardless of the training protocol.

No significant main effects of Group were detected for any variable (all *p* > 0.05), suggesting a comparable overall effectiveness of the HIIT and PHA protocols when data were averaged across the entire observation period.

In contrast, significant Time × Group interactions were found for body mass, BMI, sum of skinfolds, waist circumference, and endomorphy (*p* < 0.05–0.001), indicating different temporal patterns of adaptation between the two training protocols. Conversely, no significant interactions were observed for hip circumference, mesomorphy, or ectomorphy, suggesting similar longitudinal responses between groups for these variables.

Overall, the results indicate that both HIIT and PHA were associated with improvements in body composition, although they differed in the temporal pattern of adaptation for several key variables. The full results of the linear mixed-effects models are reported in Table 6.

The significant Time × Group interactions indicate that, although both protocols produced similar overall improvements, HIIT induced faster and greater reductions in adiposity-related variables, whereas PHA promoted a more progressive, muscle-oriented pattern of adaptation. From a practical perspective, these preliminary trajectories may inform hypotheses for supervised settings; however, confirmation in randomized controlled trials is required.

At the end of the intervention (T3), subjective psychophysical well-being was assessed using a questionnaire including three domains: psychological well-being, physical/functional well-being, and perceived performance improvement. Between-group comparisons showed no statistically significant differences in psychological well-being (*p* = 0.225) or physical/functional well-being (*p* = 0.415). In contrast, the perceived performance improvement domain differed significantly between groups, with higher scores in the HIIT group compared with the PHA group (*p* < 0.001).

Overall, both protocols were associated with a positive perception of psychophysical well-being, while HIIT was perceived as more effective in improving physical performance.

Detailed questionnaire domain scores and statistical comparisons are presented in Table 7.

## 4. Discussion

The present pilot study compared the effects of 12 weeks of HIIT and PHA training on body composition, assessed using standardized ISAK anthropometry, and on subjective psychophysical well-being in moderately active young adults. The main findings suggest that both protocols were associated with favorable within-group changes in anthropometric indicators over time; however, given the non-randomized design, these results should be interpreted as preliminary and non-causal. To our knowledge, this is among the first longitudinal studies comparing HIIT and PHA using standardized ISAK anthropometry while integrating perceived well-being outcomes in a real-world personal training setting.

In interpreting these findings, it is important to consider potential sex-related physiological and psychological differences that may influence responses to structured exercise protocols such as HIIT and PHA. Men and women differ in body fat distribution, hormonal milieu, muscle fiber composition, and neuromuscular recruitment patterns, all of which may modulate anthropometric adaptations and somatotype components [23]. Furthermore, affective and perceptual responses to high-intensity or circuit-based exercise may vary between sexes, potentially influencing subjective psychophysical outcomes [24]. Although the present pilot study was not powered to detect sex-specific effects and no stratified analyses were performed, these considerations provide an important framework for interpreting adaptation patterns and should be explored in future adequately powered randomized controlled trials.

### 4.1. Effects on Body Composition

Both groups showed significant reductions in skinfolds, BMI, and waist and hip circumferences, suggesting an overall improvement in subcutaneous adiposity. These findings are consistent with the literature indicating that high-intensity and circuit-based training programs can induce significant improvements in body composition even in the absence of structured dietary interventions [3,4,25,26].

Between-group comparisons revealed greater reductions in selected skinfolds in the HIIT group, particularly at the triceps and supraspinale sites. Although Time × Group interactions suggest different trajectories, baseline imbalance and selection bias may contribute to between-group differences. Therefore, these findings should be confirmed in randomized controlled trials. This result is in line with the physiological mechanisms typically associated with high-intensity interval training, including high metabolic stress, increased post-exercise oxygen consumption (EPOC), and activation of molecular pathways involved in lipid metabolism and mitochondrial biogenesis [4,5]. Previous studies have shown that HIIT can lead to significant fat mass reductions in relatively short timeframes, even with lower training volumes than moderate-intensity continuous exercise [2,3].

In contrast, the PHA protocol produced more pronounced increases in arm circumferences and mesomorphic somatotype, suggesting a favorable muscular adaptation. This finding is consistent with the structure of PHA, which involves a systematic alternation between upper- and lower-body exercises, promoting continuous muscular engagement and sustained hemodynamic demand without the extreme intensity peaks typical of HIIT [8]. Previous and more recent studies on total-body circuit training have reported concurrent improvements in muscular strength and aerobic capacity, with a generally favorable tolerability profile [6,7,27].

### 4.2. Longitudinal Analysis and Temporal Patterns

The linear mixed-effects model revealed a significant main effect of time for most anthropometric variables, indicating a progressive improvement in body composition regardless of the training protocol. The absence of a main group effect suggests that, when averaged over the entire observation period, HIIT and PHA showed comparable overall patterns of change.

However, the presence of significant Time × Group interactions for BMI, body mass, sum of skinfolds, and endomorphy indicates that the two protocols induced different temporal patterns of adaptation. This supports the hypothesis that HIIT and PHA can elicit qualitatively different physiological responses, even when overall improvements are similar. Such differences are consistent with previous literature showing that training programs with different combinations of intensity, volume, and work–rest distribution lead to distinct adaptive trajectories over time [5,25]. Experimental research has also demonstrated that exercise can reduce adiposity and improve anthropometric parameters even in the absence of large changes in total body mass [28], emphasizing the importance of training structure and quality in shaping adaptation patterns. Moreover, it has been widely documented that manipulating interval intensity, recovery duration, and total training volume substantially alters cardiometabolic responses, resulting in protocol-specific adaptations [29,30].

Together, these findings suggest that although both HIIT and PHA improve overall body composition, the physiological pathways and temporal dynamics through which these adaptations occur differ substantially between the two methods.

### 4.3. Psychophysical Well-Being

Both protocols were associated with a positive perception of psychological and physical/functional well-being, with no significant differences between groups. This finding aligns with evidence showing that well-structured and supervised exercise programs improve perceived well-being and quality of life, even without extreme training intensities [1,31]. Systematic reviews further indicate that regular physical activity is associated with significant improvements in quality of life and subjective well-being in adults and older populations, regardless of baseline fitness level [32]. In addition, circuit-based and combined training programs have been linked to improved perceived quality of life in working adults, suggesting that integrated exercise approaches can positively affect both physiological and psychosocial outcomes [33].

However, the HIIT group reported significantly higher scores in the perceived performance improvement domain. This finding is consistent with studies showing that HIIT can enhance both quantitative and qualitative aspects of physical performance, particularly in young and physically active populations [9,34]. The literature also indicates that although HIIT may elicit high levels of perceived exertion, it can still generate comparable or even favorable affective and enjoyment responses when appropriately structured [12].

These findings are in line with recent methodological perspectives emphasizing the importance of integrating perceptual, experiential, and awareness-based measures when evaluating training interventions, especially when the goal is to assess the sustainability and quality of the exercise experience, not just performance outcomes [19].

Importantly, psychophysical outcomes were assessed using an exploratory, non-validated questionnaire administered only at T3. Therefore, reliability (internal consistency/test–retest), construct validity, and sensitivity to change cannot be assumed. As a consequence, the observed between-group difference in perceived performance improvement may partly reflect measurement-related limitations (e.g., response bias or differential interpretation of items), in addition to potential protocol-related effects. These perceptual findings should therefore be considered preliminary and hypothesis-generating, and future studies should adopt validated instruments and/or repeated measurements over time to strengthen inference.

### 4.4. Role of Nutritional Control and Confounding Factors

Although participants were instructed to maintain their habitual diets and dietary intake was monitored through food diaries, strict nutritional control was not feasible. Consequently, the influence of dietary variation on body composition changes cannot be fully excluded. However, the literature suggests that, in real-world settings, structured exercise programs can induce meaningful improvements in body composition even without stringent dietary interventions, particularly in moderately active individuals [1,3,35]. Recent reviews also indicate that regular exercise is associated with fat mass reduction and improved body composition even in the absence of severe dietary restriction [36].

### 4.5. Study Limitations

Several limitations should be acknowledged. First, the small sample size and the non-randomized group allocation limit the generalizability of the findings and preclude strong causal inference, as selection bias cannot be excluded. Second, the absence of objective performance measures (e.g., VO_2_max or maximal strength) prevents a full integration of anthropometric changes with functional adaptations. Occasional individualized exercise substitutions were allowed for feasibility and safety reasons; although limited in number, these modifications may have introduced minor variability in the training stimulus. Furthermore, the psychophysical well-being questionnaire was administered only at the end of the intervention and was not formally validated and therefore should be considered exploratory. Finally, the sample size did not allow sex-stratified inferential analyses; consequently, potential sex-specific adaptation patterns could not be statistically examined.

### 4.6. Practical Implications and Future Perspectives

Despite the methodological limitations inherent to the pilot and non-randomized design, the present findings provide preliminary insights for supervised personal training contexts. Within-group improvements in anthropometric indicators were observed in both HIIT and PHA, with distinct adaptation patterns emerging over time. However, these results should not be interpreted as definitive evidence of efficacy or superiority of one protocol over the other.

Feasibility and implementation considerations must also be interpreted cautiously. Although adherence to prescribed training intensity was objectively monitored via heart rate recordings, structured indicators of attendance, compliance rates, and adherence to lifestyle instructions were not systematically quantified. Therefore, the generalizability of feasibility conclusions—particularly in broader, unsupervised, or non-controlled environments—cannot be definitively established based on the present data.

Future studies employing randomized allocation, larger samples, structured adherence reporting, objective performance assessments, and tighter control of potential confounding variables are required to confirm and extend these preliminary observations.

## 5. Conclusions

This pilot study compared the effects of 12 weeks of HIIT and PHA training on body composition and psychophysical well-being in moderately active young adults. Within-group improvements in anthropometric indicators were observed over time in both protocols, with distinct adaptation patterns between HIIT and PHA. Although no overall superiority emerged, the findings suggest that different training structures may be associated with specific morphological and perceptual responses. However, given the non-randomized design and absence of a non-exercise control group, these results should be considered preliminary and not interpreted as evidence of causal relationships. Future randomized controlled trials with larger samples and stricter methodological control are required to confirm and extend these observations.

## Figures and Tables

**Table 1:** Baseline (T0) anthropometric and demographic characteristics of the HIIT and PHA groups, including between-group comparisons.

Variable	HIIT (*n* = 12)	PHA (*n* = 12)	*p*-Value	Test
Sex (M/F)	5/7	7/5	0.419	Fisher exact
Age (years)	30.42 ± 3.37	31.42 ± 3.65	0.493	Welch
Triceps skinfold (mm)	15.14 ± 6.50	14.15 ± 4.81	0.366	Welch
Subscapular skinfold (mm)	11.65 ± 4.16	11.61 ± 6.36	0.684	Welch
Biceps skinfold (mm)	5.98 ± 2.98	6.13 ± 3.77	0.575	Mann–Whitney U
Suprailiac skinfold (mm)	14.02 ± 5.53	15.67 ± 5.43	0.959	Welch
Supraspinale skinfold (mm)	11.35 ± 4.67	12.54 ± 5.74	0.828	Welch
Abdominal skinfold (mm)	19.11 ± 9.10	20.61 ± 7.29	0.597	Welch
Thigh skinfold (mm)	19.32 ± 6.02	17.47 ± 7.40	0.115	Welch
Calf skinfold (mm)	12.09 ± 4.98	9.92 ± 5.63	0.069	Mann–Whitney U
Arm circumference, relaxed (cm)	29.68 ± 2.82	29.92 ± 3.43	0.910	Welch
Arm circumference, flexed and contract (cm)	30.53 ± 2.95	30.88 ± 3.66	0.947	Welch
Waist circumference (cm)	79.26 ± 12.68	83.90 ± 13.22	0.576	Welch
Hip circumference (cm)	97.98 ± 7.53	99.53 ± 6.26	0.998	Welch
Thigh circumference (cm)	49.92 ± 7.63	51.80 ± 4.35	0.996	Mann–Whitney U
Calf circumference (cm)	36.42 ± 2.79	37.23 ± 3.23	0.603	Welch
Endomorphy	3.85 ± 1.27	3.92 ± 1.32	0.602	Welch
Mesomorphy	5.04 ± 1.17	4.58 ± 1.32	0.370	Welch
Ectomorphy	1.66 ± 1.29	1.68 ± 1.04	0.603	Mann–Whitney U
BMI (kg·m^−2^)	24.21 ± 3.69	24.73 ± 3.07	0.942	Welch

Values are presented as mean ± standard deviation. Between-group comparisons were conducted using Welch’s *t*-test when normality were met, or Mann–Whitney U test when appropriate. Sex distribution was compared using Fisher’s exact test. Statistical significance was set at *p* < 0.05.

**Table 2:** Physiological parameters monitored during training sessions and their application.

Parameter	Unit	Collection Method	Frequency	Application
Heart rate	bpm	Polar H9^®^ chest strap	Continuous	Intensity monitoring
% HR_max	%	Individual calculation	Continuous	HIIT and PHA target zones
Time in intensity zones	min	Polar Flow application	Each session	Protocol adherence
Energy expenditure (estimate)	kcal	Polar proprietary algorithm	Continuous	Descriptive indicator

**Table 3:** Structure of the HIIT protocol.

Phase	Exercise	Work Duration	Rest Duration	Intensity (% HR_max)	Equipment	Notes
Warm-up	Brisk walking/Cycling	10 min	—	50–60%	Treadmill/Bike	General activation
Block 1	Battle rope	30 s	30 s	80–95%	Battle rope	High intensity
Block 2	Modified burpees	30 s	30 s	80–95%	—	Bodyweight
Block 3	Kettlebell swing	30 s	30 s	80–95%	Kettlebell	8–16 kg
Block 4	TRX row	30 s	30 s	80–95%	TRX	Adjustable angle
Block 5	Stationary bike sprint	30 s	30 s	85–95%	Bike	Maximal effort
Block 6	Jump rope	30 s	30 s	85–95%	Rope	Cardiometabolic
Inter-round recovery	—	—	60 s	60–70%	—	4 total rounds
Cool-down	Slow walking	10 min	—	50–60%	Treadmill	Heart rate reduction

**Table 4:** Structure of the PHA training protocol.

Phase	Exercise	Work Duration	Rest Duration	Target Intensity (% HR_max)	Equipment	Notes
Warm-up	Brisk walking/Cycling	10 min	—	50–60%	Treadmill/Bike	General activation
Block 1	Push-up/Chest press	40 s	20 s	70–80%	Bodyweight/Bench	Upper body
Block 2	Squat/Leg press	40 s	20 s	70–80%	Bodyweight/Machine	Lower body
Block 3	TRX row/Lat pulldown	40 s	20 s	70–80%	TRX/Lat machine	Upper body
Block 4	Alternating lunges	40 s	20 s	70–80%	Bodyweight	Lower body
Block 5	Shoulder press	40 s	20 s	70–80%	Dumbbells	Upper body
Block 6	Hip hinge/Romanian deadlift	40 s	20 s	70–80%	Barbell/Dumbbells	Lower body
Inter-round recovery	—	—	60 s	60–70%	—	4 total rounds
Cool-down	Slow walking/Mobility	10 min	—	50–60%	Treadmill	Heart rate reduction

**Table 5:** Within-group (T0–T3) and between-group (Δ) statistical comparisons for anthropometric and body composition variables.

Variable	HIIT (Within-Group)	*p*	ES	PHA (Within-Group)	*p*	ES	Between Groups (Δ)	*p*	ES
Triceps skinfold (mm)	Wilcoxon	**0.0022**	−1.000	Paired *t*-test	**0.0010**	−1.277	Mann–Whitney	**0.0089**	−0.632
Subscapular skinfold (mm)	Paired *t*-test	**0.0010**	−1.281	Paired *t*-test	0.0770	−0.563	Welch *t*-test	0.0809	−0.747
Biceps skinfold (mm)	Paired *t*-test	0.3276	−0.296	Wilcoxon	**0.0412**	−1.000	Mann–Whitney	1.0000	−0.007
Suprailiac skinfold (mm)	Wilcoxon	**0.0050**	−1.000	Paired *t*-test	**<0.0001**	−2.553	Mann–Whitney	0.0521	−0.472
Supraspinale skinfold (mm)	Paired *t*-test	**0.0013**	−1.234	Wilcoxon	**0.0072**	−1.000	Mann–Whitney	**0.0440**	−0.486
Abdominal skinfold (mm)	Paired *t*-test	**0.0077**	−0.992	Paired *t*-test	**0.0006**	−1.478	Welch *t*-test	0.1209	−0.666
Thigh skinfold (mm)	Paired *t*-test	**0.0004**	−1.627	Wilcoxon	**0.0022**	−1.000	Mann–Whitney	0.2453	−0.292
Calf skinfold (mm)	Paired *t*-test	**0.0002**	−1.822	Wilcoxon	**0.0098**	−1.000	Mann–Whitney	0.1265	−0.375
Arm circumference (relaxed, cm)	Paired *t*-test	0.4273	−0.241	Paired *t*-test	**0.0022**	+1.095	Welch *t*-test	**0.0102**	−1.109
Arm circumference (flexed, cm)	Paired *t*-test	0.1587	−0.430	Paired *t*-test	**0.0047**	+0.949	Welch *t*-test	**0.0025**	−1.485
Waist circumference (cm)	Paired *t*-test	**0.0001**	−2.031	Paired *t*-test	**0.0001**	−1.938	Welch *t*-test	0.6626	+0.126
Hip circumference (cm)	Paired *t*-test	**0.0008**	−1.342	Wilcoxon	**0.0106**	−0.955	Mann–Whitney	0.8812	−0.083
Thigh circumference (cm)	Wilcoxon	**0.0392**	−0.591	Paired *t*-test	**0.0305**	+0.707	Mann–Whitney	**0.0030**	−0.833
Calf circumference (cm)	Paired *t*-test	**0.0316**	+0.679	Paired *t*-test	**0.0014**	+1.246	Welch *t*-test	0.3821	−0.454
Endomorphy	Paired *t*-test	**0.0037**	−1.082	Paired *t*-test	**0.0010**	−1.250	Welch *t*-test	0.9186	+0.040
Mesomorphy	Paired *t*-test	**0.0022**	+1.149	Wilcoxon	**0.0342**	+0.682	Mann–Whitney	**0.0078**	+0.639
Ectomorphy	Paired *t*-test	**0.0029**	+1.098	Paired *t*-test	**0.0006**	+1.493	Welch *t*-test	0.1158	−0.684
BMI (kg·m^−2^)	Paired *t*-test	**0.0003**	−1.547	Paired *t*-test	**0.0001**	−2.078	Welch *t*-test	0.3377	+0.467

Statistically significant *p*-values are highlighted in bold (*p* < 0.05). ES = effect size (Cohen’s *d* for parametric tests, *r* for non-parametric tests).

**Table 6:** Linear mixed-effects model results for longitudinal changes (Time, Group, and Time × Group effects).

Variable	Time Effect	Group Effect	Time × Group	Interpretation
Body mass	*p* < 0.001	n.s.	*p* < 0.001	Significant reduction over time with different temporal patterns between protocols
BMI	*p* < 0.001	n.s.	*p* < 0.01	Overall decrease over time with protocol-specific trajectories
Sum of skinfolds (Σ8)	*p* < 0.001	n.s.	*p* < 0.01	Progressive reduction in subcutaneous adiposity with different time courses
Waist circumference	*p* < 0.001	n.s.	*p* < 0.05	Significant decrease over time with differential temporal response
Hip circumference	*p* < 0.001	n.s.	n.s.	Similar reduction over time in both groups
Endomorphy	*p* < 0.01	n.s.	*p* < 0.05	Decrease over time with different adaptation patterns
Mesomorphy	*p* < 0.05	n.s.	n.s.	Increase over time, comparable between groups
Ectomorphy	n.s.	n.s.	n.s.	No significant longitudinal changes

Note. n.s. = not significant.

**Table 7:** Psychophysical well-being questionnaire scores at T3.

Domain	HIIT (Mean ± SD)	PHA (Mean ± SD)	Statistical Test	*p*-Value
Psychological well-being	3.42 ± 0.73	3.75 ± 0.62	Mann–Whitney U	0.225
Physical/functional well-being	4.04 ± 0.40	3.83 ± 0.69	Mann–Whitney U	0.415
Perceived performance improvement	4.63 ± 0.43	3.04 ± 0.54	Mann–Whitney U	<0.001

## Data Availability

All data generated or analyzed during this study have been included within the manuscript.
